# Citations increase with manuscript length, author number, and references cited in ecology journals

**DOI:** 10.1002/ece3.2505

**Published:** 2016-10-05

**Authors:** Charles W. Fox, C. E. Timothy Paine, Boris Sauterey

**Affiliations:** ^1^ Department of Entomology University of Kentucky Lexington KY USA; ^2^ Biological and Environmental Sciences University of Stirling Stirling UK

**Keywords:** bibliometrics, citation analysis, journal guidelines, research impact, scientific publication

## Abstract

Most top impact factor ecology journals indicate a preference or requirement for short manuscripts; some state clearly defined word limits, whereas others indicate a preference for more concise papers. Yet evidence from a variety of academic fields indicates that within journals longer papers are both more positively reviewed by referees and more highly cited. We examine the relationship between citations received and manuscript length, number of authors, and number of references cited for papers published in 32 ecology journals between 2009 and 2012. We find that longer papers, those with more authors, and those that cite more references are cited more. Although paper length, author count, and references cited all positively covary, an increase in each independently predicts an increase in citations received, with estimated relationships positive for all the journals we examined. That all three variables covary positively with citations suggests that papers presenting more and a greater diversity of data and ideas are more impactful. We suggest that the imposition of arbitrary manuscript length limits discourages the publication of more impactful studies. We propose that journals abolish arbitrary word or page limits, avoid declining papers (or requiring shortening) on the basis of length alone (irrespective of content), and adopt the philosophy that papers should be as long as they need to be.

## 1 Introduction

Scholarly papers are the primary medium through which scientific researchers communicate ideas and research outcomes to their peers. The number of papers published in the scholarly scientific literature has been increasing exponentially, at a rate of approximately 3% per year, since 1980 (Bornmann & Mutz, 2015). This growth rate has been slightly higher in ecology and evolution than in other biological disciplines (Pautasso, 2012). At many journals, submissions are growing at a faster pace than are the page allocations necessary to publish those submissions (Fox & Burns, 2015). This disparity drives down acceptance rates (Fox & Burns, 2015; Fox, Burns, & Meyer, 2016; Wardle, 2012), but also puts pressure on editors to allocate fewer pages to each published manuscript so that journals can publish more papers while staying within contractual page budgets.

Most top impact factor ecology journals indicate a preference or requirement for short manuscripts (25 of the 32 journals in Appendix Table A1). Some state clearly defined word limits, generally requiring manuscripts to contain fewer than 6000–8000 words, although which elements of the paper this includes (e.g., including references or just the main text), and the degree to which these are guidelines versus absolute limits, varies among journals. Other journals have less specific word or page limits but nonetheless emphasize that shorter papers are preferable. *Ecology*, for example, warns that “many manuscripts submitted to *Ecology* are rejected without review for being overly long” and *Functional Ecology* notes that “preference is given to shorter, more concise papers” (Appendix Table A1). Also, because evaluations of researcher performance commonly consider publication counts more than publication length when quantifying researcher impact, authors may choose to split complex studies into smaller publication units to increase their number of publications. Journals and authors thus commonly prefer shorter papers. How does this influence the impact of papers?

The perspective that short manuscripts have greater impact is likely driven by the observation that the highest profile journals, such as *Science* and *Nature* for general science, or *Ecology Letters* within ecology, publish relatively short articles. Evidence also suggests that social media attention is greater for shorter paper (Haustein, Costas, & Larivière, 2015). However, few research papers receive attention on social media (in contrast to editorials and news items; Haustein et al., 2015), especially if published outside the major multidisciplinary journals (Zahedi, Costas, & Wouters, 2014), and social media attention (except for Mendeley) generally only weakly correlates with citations received in the scholarly literature (Haustein et al., 2014). Evidence in a variety of academic fields indicates that, within journals, *longer* papers are both more positively reviewed by referees (Card & DellaVigna, 2012) and more highly cited (Ball, 2008; Falagas, Zarkali, Karageorgopoulos, Bardakas, & Mavros, 2013; Haustein et al., 2015; Leimu & Koricheva, 2005b; Perneger, 2004; Robson & Mousquès, 2014; Schwarz & Kennicutt, 2004; Vanclay, 2013; Xiao, Yuan, & Wu, 2009). Many research projects produce complex data that does not lend itself to concise presentation of a single or simple message. It is thus likely that longer papers contain more ideas and a greater diversity of results, which provides more opportunity for citation (Leimu & Koricheva, 2005b), and thus have more diverse and possibly greater impact on the scientific community.

The objective of this study was to examine the relationships between citations received, a proxy for academic impact, and manuscript length at major ecology journals. However, manuscript length covaries positively with a variety of other features that have been shown to predict citation frequency. In particular, papers with more authors are commonly better cited (Leimu & Koricheva, 2005a,b; Schwarz & Kennicutt, 2004; Borsuk, Budden, Leimu, Aarssen, & Lortie, 2009; Webster, Jonason, & Schember, 2009; Gazni & Didegah, 2011; Didegah & Thelwall, 2013; Robson & Mousquès, 2014; Haustein et al., 2015; Larivière, Gingras, Sugimoto, & Tsou, 2014; but see Stremersch, Verniers, & Verhoef, 2007; Rao, 2011). It is possible that this occurs because more authors on a paper leads to more self‐citation and/or citation by colleagues and collaborators, but it is more likely that collaborative projects present more diverse data and ideas and are of higher quality (Katz & Martin, 1997). Also, longer papers tend to cite more references (Abt & Garfield, 2002) and papers that cite more references tend to be better cited (Webster et al., 2009; Mingers & Xu, 2010; Rao, 2011; Bornmann, Schier, Marx, & Daniel, 2012; Robson & Mousquès, 2014; Ale Ebrahim, Ebrahimian, Mousavi, & Tahriri, 2015; Haustein et al., 2015; review of earlier work in Alimohammadi & Sajjadi, 2009). There is even evidence that papers with longer abstracts are better cited (Weinberger, Evans, & Allesina, 2015), possibly because more data‐ or idea‐rich papers have longer abstracts, or just because longer abstracts touch on more points and are thus more likely attract reader interest. These various relationships make it difficult to determine causality in analyses of how manuscript length predicts citation frequency.

We examine the relationships between citations received and manuscript length, number of authors, and number of references cited for papers published in 32 ecology journals between 2009 and 2012 (inclusive). We find that, within journals, longer papers, papers with more authors, and papers with more references are better cited. We argue that the preference by journal editors for short papers (and short abstracts), and journal‐imposed limits on manuscript length, are likely to reduce the scientific impact of published articles.

## 2 Methods

### 2.1 Dataset

Citation data were retrieved from Web of Science for 32 ecology journals between 29 September and 2 October 2014 (Monday–Thursday). Extraction of citation data was completed before the weekly update of the Web of Science database that occurred on 2 October, and thus data are from the same Web of Science update for all journals. Citation counts are an imperfect metric of manuscript impact. They do not capture influence on practitioners (Stremersch et al., 2007) and can covary with many variables unrelated to manuscript quality or influence, such as author reputation (Mingers & Xu, 2010). However, citations covary with other measures of scientific influence (Mingers & Xu, 2010) and article downloads (Perneger, 2004; although this relationship varies among journals and disciplines, Bollen, Van de Sompel, Smith, & Luce, 2005), and they can be objectively quantified.

The journals were chosen from the list of all journals that received an impact factor and were categorized as ecology journals by Thomson Reuters in 2013. We included journals based on the following criteria. The journal must have (i) published at least 400 research articles in the 4‐year window of this study, (ii) had a 2013 two‐year impact factor of 2.5 or greater (as low impact factors indicate that many articles go uncited), and (iii) publish primarily research papers (e.g., we exclude the *Annual Review* and *Trends* series). Limiting our analyses to journals with an impact factor >2.5 could introduce bias into measures of the relationship between manuscript length and citations because it excludes a large number of low citation papers. However, journals with higher impact factors are those under the most pressure to publish shorter papers (because they receive far more submissions than they can publish). Also, relationships described below (in 3) are consistent across all journals in our dataset, including those with higher and lower impact factors. Nonetheless, we must be cautious extrapolating from our analysis of journals with higher impact factors to the broader ecological literature. We also excluded journals that publish primarily in a language other than English (e.g., *Interciencia*), those with a primarily methodological focus (e.g., *Molecular Ecology Resources*) and those with a primary focus in another discipline than ecology (e.g., *Ecological Engineering*,* Ecological Economics* and *Ecology and Society*). These criteria yielded 26,539 articles.

We include in analyses all regular papers (those identified as “articles” in Web of Science) published between 2009 and 2012 (inclusive); we exclude all papers not tagged as an “article,” which includes reviews, editorials, and a variety of other nonstandard manuscript types. We chose these years, 2009–2012, rather than older publication years (which had more time to accumulate citations), so that our analyses to reflect the current state of ecology publishing. We also exclude all papers that were categorized as an “article” but that cited no references, had titles of fewer than three words, were fewer than two pages long, had more than 200 references, or had abstracts of fewer than 10 words. These were papers likely to be miscategorized by Web of Science. The final dataset includes 26,088 articles.

### 2.2 Analyses

As an initial exploration of the data, we performed an ANCOVA predicting the number of citations an article received as a function of its page length and the journal in which it was published. These factors were allowed to interact to determine the degree to which the citation–page length relationship varied among journals. We also included year of publication, as articles published in early 2009 had 5.8 years to accumulate citations, whereas those published in late 2012 had only 1.8 years to do so. We note that citations obtained by a manuscript soon after publication are predictive of the citations it will obtain later (Adams, 2005). Thus, the form of the ANCOVA was Number_of_citations ~ Year + Page_length * Journal.

Page length, however, covaries with other factors, including the number of authors and number of references, that may also influence an article's impact on the scientific community (Figure 2). Therefore, we next built a mixed‐effect model to assess the relative importance of page length, the number of authors, and the number of references on the number of citations received by an article, together with all their interactions. Year and journal were included as random effects. We also allowed for random variation in the three main effects among journals. Thus, the form of the mixed‐effect model was Number_of_citations ~ Number_of_references * Number_of_pages * Author_count + (1|Year) + (Number_of_pages + Number_of_references + Author_count|Journal), where the brackets around the last two terms indicate that they are random effects, with the grouping factors to the right of the vertical bar. Note that it was not possible to include “page count excluding references” in our models because we only have access to the total page count and number of references, and not how many pages are allocated to each manuscript's reference section. All fixed effects were standardized to a mean of zero and standard deviation of one to allow comparisons of their relative contributions to the number of citations received. In both analyses, the number of citations (+1), the number of pages, and the author count were log‐transformed to reduce heteroscedasticity. Year was included as a factor with four levels to allow free variation in citations received among years. Confidence intervals and *p*‐values were estimated with 1000 parametric bootstrap replicates. Analyses were performed in the R language and environment version 3.2.3. The mixed‐effect model was implemented using the lme4 package (Bates, 2005).

## 3 Results

### 3.1 Longer papers are better cited than shorter papers

**Figure 1:**
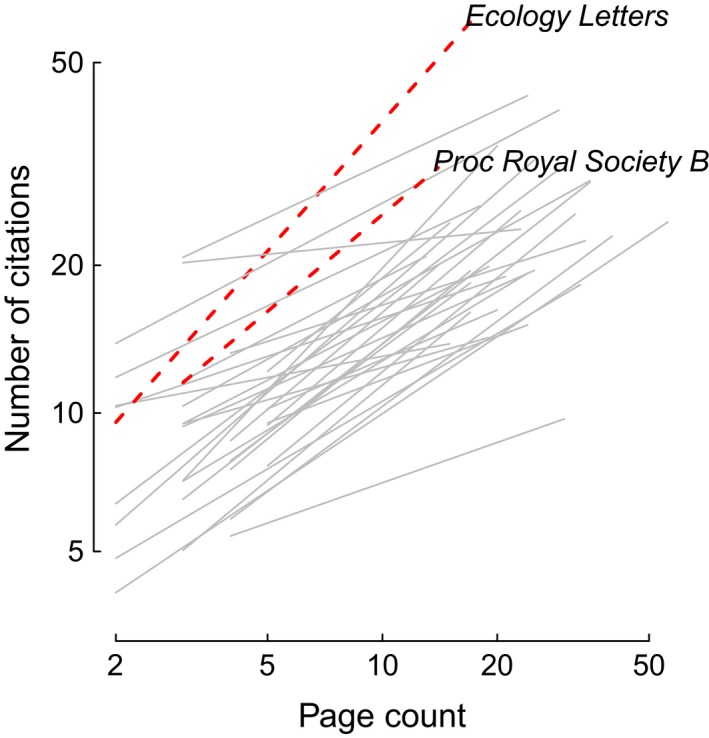
The relationship between total citations received and manuscript length for papers published 2009–2012 in 32 ecology journals. Lines represent the predictions for all journals from the ANCOVA model. Journals mentioned in the text are denoted with red‐dashed lines and are labeled.

Across all journals, longer papers were consistently more highly cited than shorter papers (Figure 1). The slope of the relationships between citations and page length varied substantially among journals, as would be expected due to variation in manuscript formatting, mean paper lengths, and citation counts among journals (See Appendix Table A1). It is notable that the relationships between citations and page count were particularly steep for the shorter‐format journals (e.g., *Ecology Letters* and *Proceedings of the Royal Society of London B*; Figure 1).

However, this relationship could be a consequence of covariance between manuscript length and other variables that influence citations. In particular, the number of references cited by papers and the number of authors on papers have both been demonstrated to influence citation rates.

### 3.2 Papers that cite more references and have more authors are better cited

**Figure 2:**
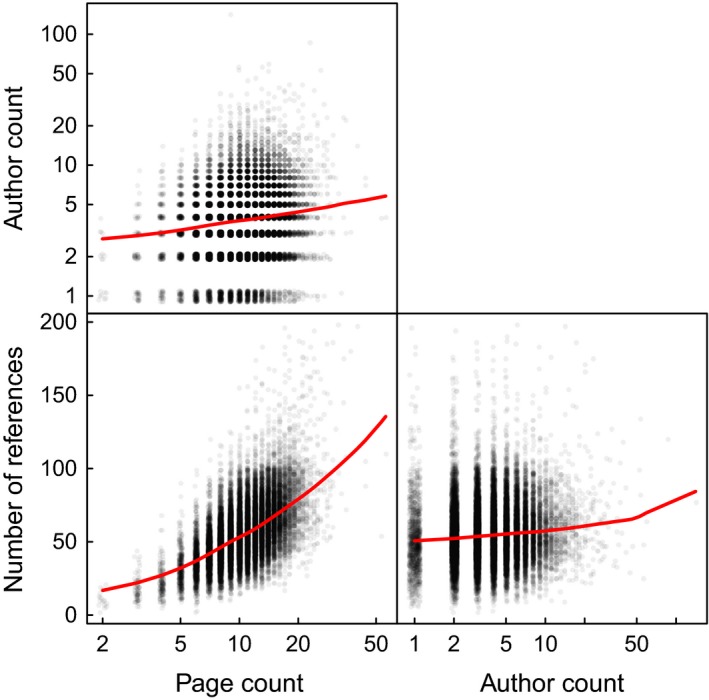
Scatterplot matrix showing intercorrelations of predictor variables. Points have been jittered for legibility. Red lines are smoothed lowess regressions. Number of pages and number of authors are presented on log‐transformed axes.

For ecology journals, page count, author count, and references cited all covary positively (Figure 2). Papers with more authors tend to be longer (*r*_absolute_ = .16; *p *<* *.001) and cite more references (*r*_absolute_ = .09; *p *< .001), and longer papers tend to cite more references (*r*_absolute_ = .56; *p *<* *.001). We thus used a mixed‐effect model to assess their relative contribution to citation frequency.

**Figure 3:**
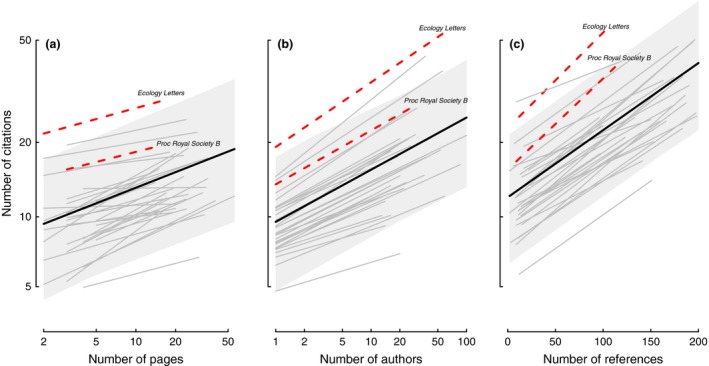
The relationship between total citations received and (a) manuscript length, (b) number of authors, and (c) number of references for papers published 2009–2012 in 32 ecology journals. Overall relationships from the mixed‐effect model are shown with heavy solid lines and confidence intervals, whereas relationships for individual journals are shown in thin lines. Lines are partial regressions after controlling for other effects in the full model presented in Table 1. Journals highlighted in Figure 1 are denoted with red‐dashed lines and are labeled. All other variables are held at their medians. Note that the X‐axes of panels (a) and (b), as well as all Y‐axes, are log‐transformed.

The model including these three variables indicated that manuscript length, author count, and references cited all covary positively with the number of citations received by an article (Figure 3, Table 1). On average, a 10% increase in page count from the median (from 10 to 11 pages) generated a 1.8% increase in the number of times an article was cited. This increase varied among journals from a high of a 3.8% increase in citations for a 10% increase in manuscript length above the median in *Behavioral Ecology and Sociobiology* to a low of just 0.1% for *Ecological Applications*—the relationship is always positive but often small. A 10% increase in author count (from a median of 4 to 4.4 authors) had a similar effect, increasing the number of times an article was cited by 1.9%. A 10% increase in the number of references in the average journal (from a median of 54 to 59.4 references) increased the number of times an article was cited by 3.3%.

**Table 1:** The influence of manuscript length (pages), the number of authors, and reference count on the number of citations received

Source	Degrees of freedom	Estimate	95% confidence interval	*p*‐value
Intercept	1	1.027	0.819 to 1.235	<.001
Number of References	1	0.047	0.040 to 0.054	<.001
Log (Page count)	1	0.022	0.014 to 0.031	<.001
Log (Author count)	1	0.043	0.038 to 0.049	<.001
Number of References × Log (Page count)	1	−0.002	−0.005 to 0.002	.174
Number of References × Log (Author count)	1	−0.008	−0.012 to −0.003	.001
Log (Page count) × Log (Author count)	1	0.005	0.000 to 0.009	.019
Log (Page count) × Log(Author count) × Number of references	1	0.005	0.002 to 0.007	<.001

The dependent variable is log(total citations received + 1), which was predicted as a function of number of references, log‐transformed number of pages, and log‐transformed number of authors, together with their interactions. The random effects were journal, which was allowed to interact with each of the main fixed effects and year. Parameter estimates are derived from the version of the model in which all numeric predictors were standardized to mean 0 and unit variance. Thus, the relative magnitudes of each estimated parameter indicate their relative importance in affecting the number of citations obtained. Confidence intervals and *p*‐values were estimated with 1000 parametric bootstrap replicates.

**Figure A1:**
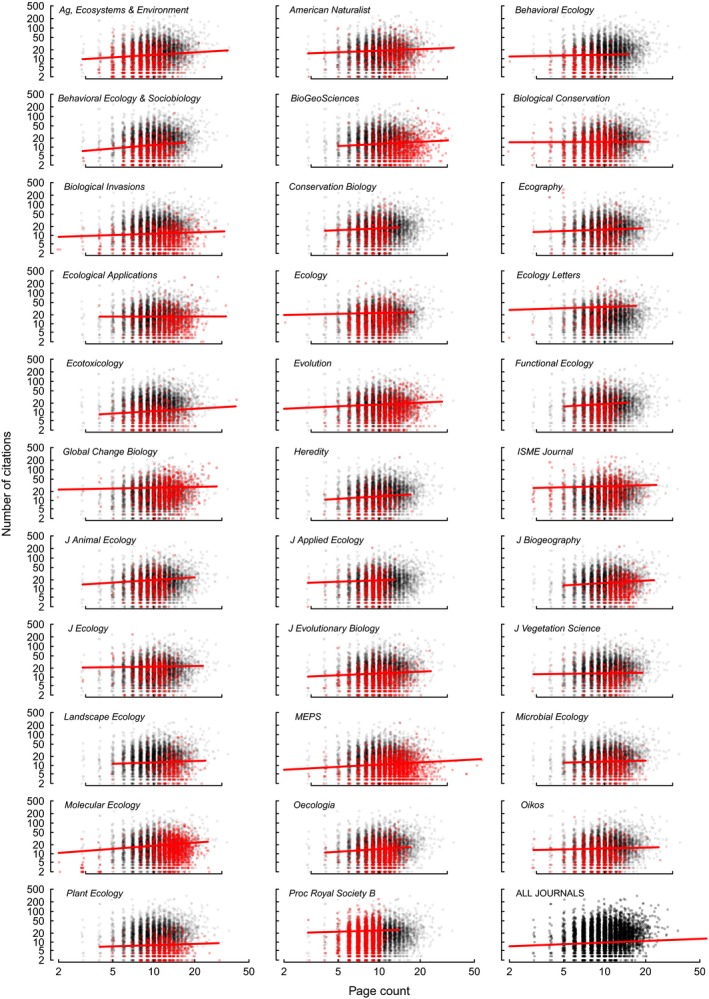
The relationship between citations received and manuscript length of papers published in 2010 in 32 ecology journals. Lines represent predictions from the mixed‐effect model, holding all other predictors constant at their medians.

Notably, the relationships between citations obtained on the one hand and page count, author count, and number of references on the other were consistently positive across all journals and years (Figure 3; see also Appendix Figure A1). The number of citations was positively correlated with page count for *all* of 32 journals, and this relationship was significantly greater than zero (*p *<* *.05) for 13 of 32 journals (Figure 3a). Moreover, the number of citations was significantly positively associated with author count and the number of references for every one of the 32 journals studied (Figure 3b, c).

## 4 Discussion

Longer research papers are, on average, more highly cited than are shorter papers across the ecology literature. This remains the case after accounting for variation in author number and references—papers with more authors and that cite more references tend to be both longer and more highly cited. Although the proportion of variance explained by each of these variables is small (as expected given the high variance in citations among papers within journals), the observed effect sizes are moderate, with each additional 10% of manuscript length increasing citations by an average of approximately 1.8% (across all journals) after controlling for other predictors.

Longer papers are probably better cited because they contain both more and a greater diversity of data and ideas (Leimu & Koricheva, 2005b). We argue that the positive relationship between citations and both author number and references cited support this hypothesis. Studies that have more authors tend to draw on a greater diversity of expertise, whether practical or intellectual (Katz & Martin, 1997), and thus present a greater diversity of ideas and/or data types, especially when collaborations are interdisciplinary. Likewise, papers likely cite more references because they have a greater diversity of arguments to support or ideas to place into context. Alternatively, a longer reference list may make a particular paper more visible, as the study will show up on search results in citation databases more frequently (Didegah & Thelwall, 2013) or it may encourage researchers that have been cited to cite the paper (i.e., tit‐for‐tat citation; Webster et al., 2009). Indeed, some people have suggested authors can increase the number of citations their papers will receive by increasing the number of references they cite (e.g., Ball, 2008; Webster et al., 2009). Papers with more authors have more individuals potentially self‐citing the manuscript (Larivière et al., 2014) and have a larger network of colleagues that may cite the paper (Borsuk et al., 2009). However, despite the potential influences of increased visibility, tit‐for‐tat citation, and self‐citation, we expect that it is the increase in citable content that drives most of the correlations with citations.

Although citations increase with page count, they increase more slowly than does page count; that is, citations per page are negatively correlated with number of pages (as observed by Stanek, 2008). This is not surprising—although papers that present more citable material should be cited in a larger number of subsequent papers, each subsequent citation is only counted once regardless of how many distinct points in the original study are referenced by each citing paper. So, a longer paper cited for two or more distinct points in a single citing paper counts as the same number of citations as does a shorter paper cited for just one point.

We cannot know for any published study if a longer version of that same article would have received more citations, or whether the published versions of studies are, on average, the length that maximizes their quality and impact. However, multiple lines of evidence indicate that imposing arbitrary length limits on papers has a negative impact. In economics, the adoption of a policy imposing strict manuscript length limits led to a significant decrease in submissions (rather than an equivalent number of shorter submissions) from certain subfields, notably those for which papers tended to be longer (Card & DellaVigna, 2012, 2014). Although authors in these subfields may have just preferred (or had more opportunity) to switch journals rather than spend time revising their manuscripts, they may also be unable to shorten their manuscripts without significant (and unacceptable) losses of content and quality. The economics literature also provides evidence that authors massage their submissions to circumvent page limits imposed by top impact journals; although some authors cut text to conform to journal requirements, others change fonts, spacing and margins to force content to fit into journal page limits (Card & DellaVigna, 2012, 2014). The now widespread use of supplemental material, compared to just 10–15 years ago (Borowski, 2011; Kenyon & Sprague, 2014), also signals a problem. Much of this growth in use of supplementary material certainly reflects authors making available information they might previously have never published, which is clearly a benefit to science. However, supplementary material is more common and more extensive in journals that impose page limits (Pop & Salzberg, 2015), indicating that much of the content is excised from manuscripts to keep them concise (Moore & Beckerman, 2016). It is good, of course, that this information is available to readers, but supplemental material is almost always in separate documents from the main text, often lacks identifying information to link it to the study, is published online in a wide variety of (often proprietary) electronic formats, is rarely carefully evaluated by peer reviewers, is infrequently read, and has little guarantee of long‐term preservation or availability (Evangelou, Trikalinos, & Ioannidis, 2005; Williams, 2016). As Moore and Beckerman (2016) note, supplementary material is “where data and methods go to die.”

## 5 Conclusion

Across the ecology literature, longer papers are, on average, more highly cited than shorter papers. This is likely because longer papers contain more data and ideas and thus have more citable elements. This relationship has been noted previously (Leimu & Koricheva, 2005b), yet journal policies commonly indicate a preference or requirement for short papers. There is also a perception among ecologists that shorter papers are more impactful. Short papers may be quicker to read and thus read more often (Moore, 2011), and short single‐message papers may reach conclusions that are easier to recall. However, they are not as well cited as long papers.

We suggest that the adoption of arbitrary manuscript length limits discourages publication of more impactful studies, negatively impacting science. Even when such limits are unenforced, we suspect that they discourage at least some authors from giving their science the complete presentation it deserves (longer, meatier papers). We emphasize, though, that we do not argue here that simply making papers longer will increase their impact—increasing article length without a concomitant increase in scientific content would be counterproductive. The perfect length for a manuscript is that which is necessary to present all of the data and ideas that arise from the study, but not longer. We suspect (or at least hope) that most published manuscripts are near this length. But journal manuscript length policies, as publicized if not always as enforced, rarely recognize this. These policies may serve the immediate needs of the journals adopting them, but do not serve the long‐term needs of the authors or the scientific community. We propose that the scientific literature will be improved if journals abolish arbitrary manuscript word or page limits, avoid declining papers (or requiring shortening) on the basis of length alone, and adopt the philosophy that papers should be as long as they need to be (but not longer).

## Conflict of interest

None declared.

## Appendix 1

### 1.1

**Table A1:** Preferences regarding manuscript length for standard/original research papers presented in author guidelines for the 32 ecology journals included in this study (as of 1 July 2016)

Journal name	Guidelines concerning manuscript length	Web link
Agriculture, Ecosystems & Environment	None specified	https://www.elsevier.com/journals/agriculture-ecosystems-and-environment/0167-8809/guide-for-authors
The American Naturalist	“preference is for manuscripts that are approximately 21 manuscript pages or fewer of text”	http://www.journals.uchicago.edu/journals/an/instruct
Behavioral Ecology	“concise”	http://www.oxfordjournals.org/our_journals/beheco/for_authors/general.html
Behavioral Ecology and Sociobiology	“papers should not exceed 13 printed pages”	https://www.springer.com/life+sciences/behavioural/journal/265
Biogeosciences	None specified	http://www.biogeosciences.net/submission/manuscript_preparation.html
Biological Conservation	“up to 8,000 words” where “figure or table should be considered equal to 300 words”	https://www.elsevier.com/journals/biological-conservation/0006-3207/guide-for-authors
Biological Invasions	“no specific page or word limits” but “as a guide the average original paper contains approximately 8,000 words”	https://www.springer.com/life+sciences/ecology/journal/10530
Conservation Biology	“3000–6000 words” that includes “all text from the first word of the Abstract through the last word in Literature Cited”	http://onlinelibrary.wiley.com/journal/10.1111/(ISSN)1523-1739/homepage/ForAuthors.html
Ecography	None specified	http://www.ecography.org/authors/author-guidelines
Ecological Applications	“60 manuscript pages”	http://esapubs.org/esapubs/AuthorInstructions.htm
Ecology	“20‐30 manuscript pages” and “many manuscripts submitted to Ecology are rejected without review for being overly long” and “We are asking authors to submit shorter, better‐organized pieces”	http://esapubs.org/esapubs/AuthorInstructions.htm
Ecology Letters	“maximum of 5000 words”	http://onlinelibrary.wiley.com/journal/10.1111/(ISSN)1461-0248/homepage/ForAuthors.html
Ecotoxicology	None specified	https://www.springer.com/environment/journal/10646
Evolution	“7500 words of text”	http://onlinelibrary.wiley.com/journal/10.1111/(ISSN)1558-5646/homepage/ForAuthors.html
Functional Ecology	“preference is given to shorter, more concise papers” and “target length of Standard Papers is approximately 7,000 words including references”	http://www.functionalecology.org/view/0/authorGuideline.html
Global Change Biology	8,000 words	http://onlinelibrary.wiley.com/journal/10.1111/(ISSN)1365-2486/homepage/ForAuthors.html
Heredity	7,000 words excluding references	http://mts-hdy.nature.com/cgi-bin/main.plex?form_type=display_auth_instructions
ISME Journal	“5,000 words max excluding references, figures and tables”	http://www.nature.com/ismej/about/for_authors.html
Journal of Animal Ecology	“A standard paper should not normally be longer than 8500 words, including all text, references, tables and figure legends”	http://www.journalofanimalecology.org/view/0/authorGuideline.html
Journal of Applied Ecology	“should not exceed 7000 words … inclusive of all parts of the paper”	http://www.journalofappliedecology.org/view/0/authorGuideline.html
Journal of Biogeography	“should not exceed 7000 words … inclusive of abstract, main text and references”	http://onlinelibrary.wiley.com/journal/10.1111/(ISSN)1365-2699/homepage/ForAuthors.html
Journal of Ecology	“should not normally be longer than 12 printed pages”	http://www.journalofecology.org/view/0/authorGuideline.html
Journal of Evolutionary Biology	“should not typically exceed 10 printed pages”	http://onlinelibrary.wiley.com/journal/10.1111/(ISSN)1420-9101/homepage/ForAuthors.html
Journal of Vegetation Science	“typical length of ordinary papers is about 8–10 printed pages”	http://onlinelibrary.wiley.com/journal/10.1111/(ISSN)1654-1103/homepage/ForAuthors.html
Landscape Ecology	“8500 words”	https://www.springer.com/life+sciences/ecology/journal/10980
Marine Ecology Progress Series	“target: ~6000 words”	http://www.int-res.com/journals/meps/guidelines-for-meps-authors/
Microbial Ecology	None specified	https://www.springer.com/life+sciences/microbiology/journal/248
Molecular Ecology	“8000 words per paper, excluding references”	http://onlinelibrary.wiley.com/journal/10.1111/(ISSN)1365-294X/homepage/ForAuthors.html
Oecologia	“10 printed pages (equivalent to approximately 35 submitted pages)”	https://www.springer.com/life+sciences/ecology/journal/442
Oikos	None specified	http://www.oikosjournal.org/authors/author-guidelines
Plant Ecology	“6,000 words”	https://www.springer.com/life+sciences/plant+sciences/journal/11258
Proceedings of the Royal Society of London B	“levies charges for research articles which exceed 6 printed pages when published in the journal” but will “consider articles that exceed this limit, up to 10 printed pages of the journal”	http://rspb.royalsocietypublishing.org/content/author-information
